# Supplementary figures and images for: Tulp3 quantitative alleles titrate requirements for viability, brain development, and kidney homeostasis but do not suppress Zfp423 mutations in mice
Source: PLoS Genet. 2025 Oct 15;21(10):e1011911. doi: 10.1371/journal.pgen.1011911 (PMC12539727; doi:10.1371/journal.pgen.1011911)

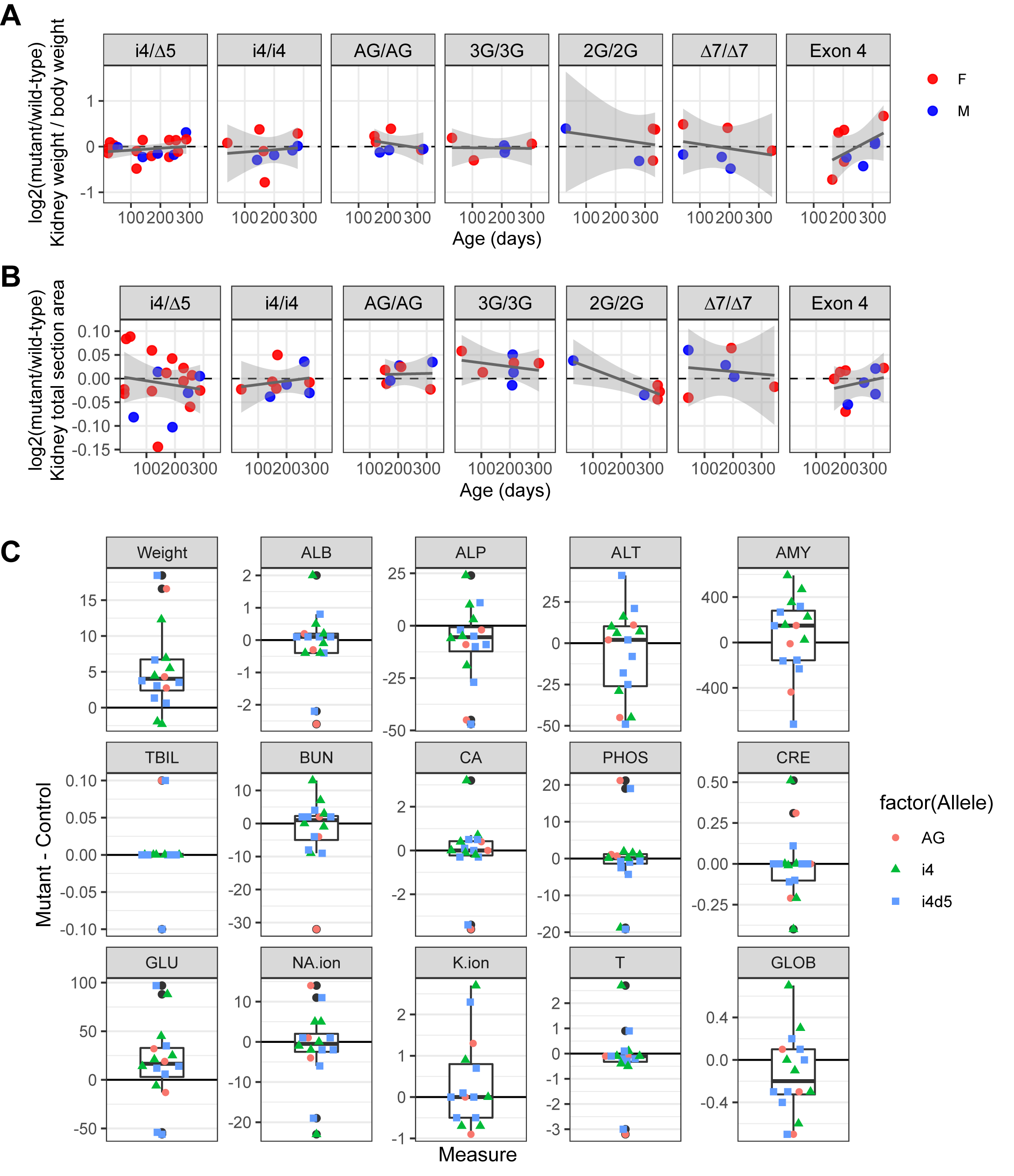

Supplement: S1 Fig — (A) Ratio of kidney weight to body weight was not significantly different between genotypes for any variant, p > 0.2. (B) Total cross-sectional area of kidney was not significantly different between genotypes for any variant, p > 0.2. (C) No striking differences in blood chemistries among 16 sex-matched littermate pairs. Standard blood chemistry panels from same-sex littermate pairs from strong Tulp3 allele combinations found no consistent differences dependent on genotype, in contrast to their body weights, which confirmed the differences shown in Fig 3. Box-and-whiskers plots with individual data points for paired sample differences (mutant – littermate control) for Albumin (ALB), Alkaline Phosphatase (ALP), Alanine Transaminase (ALT), Amylase (AMY), total Bilirubin (TBIL), Blood Urea Nitrogen (BUN), Calcium (CA), Phosphorus (PHOS), Creatinine (CRE), Glucose (GLU), Sodium (NA-ion), Potassium (K-ion), Total Protein (TP), and calculated Globulin (GLOB). (TIF) [file pgen.1011911.s013.tif]

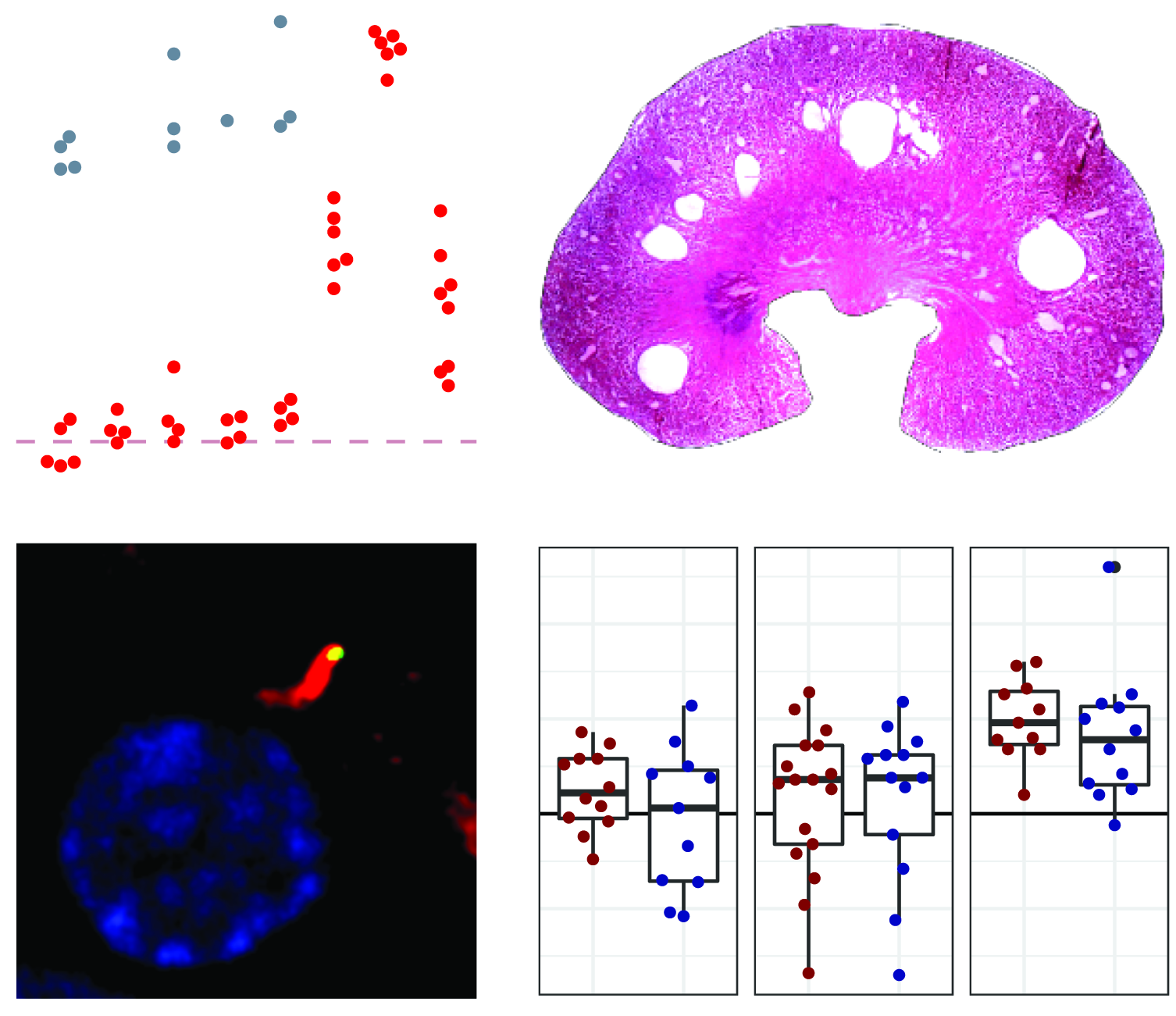

Supplement: S2 — (TIF) [file pgen.1011911.s014.tif]
